# Co-existence of Network Architectures Supporting the Human Gut Microbiome

**DOI:** 10.1016/j.isci.2019.11.032

**Published:** 2019-11-21

**Authors:** Caitlin V. Hall, Anton Lord, Richard Betzel, Martha Zakrzewski, Lisa A. Simms, Andrew Zalesky, Graham Radford-Smith, Luca Cocchi

**Affiliations:** 1 Clinical Brain Networks Group, QIMR Berghofer Medical Research Institute, Brisbane, QLD 4006, Australia; 2 School of Biomedical Sciences, University of Queensland, Brisbane, QLD 4006, Australia; 3 Gut Health Lab, QIMR Berghofer Medical Research Institute, Brisbane, QLD 4006, Australia; 4 Department of Psychological and Brain Sciences, Indiana University, Bloomington, IN 47405, USA; 5 Medical Genomics Group, QIMR Berghofer Medical Research Institute, Brisbane, QLD 4006, Australia; 6 Melbourne Neuropsychiatry Centre and Department of Biomedical Engineering, The University of Melbourne, Melbourne, VIC 3010, Australia

**Keywords:** Microbiology, Microbiome, Bioinformatics, Association Analysis

## Abstract

Microbial organisms of the human gut microbiome do not exist in isolation but form complex and diverse interactions to maintain health and reduce risk of disease development. The organization of the gut microbiome is assumed to be a singular assortative network, where interactions between operational taxonomic units (OTUs) can readily be clustered into segregated and distinct communities. Here, we leverage recent methodological advances in network modeling to assess whether communities in the human microbiome exhibit a single network structure or whether co-existing mesoscale network architectures are present. We found evidence for core-periphery structures in the microbiome, supported by strong, assortative community interactions. This complex architecture, coupled with previously reported functional roles of OTUs, provides a nuanced understanding of how the microbiome simultaneously promotes high microbial diversity and maintains functional redundancy.

## Highlights

• The gut microbiome conforms to an assortative and core-periphery mesoscale network
• Co-existing mesoscale structures support high functional redundancy
• Each community contributes to a diverse number of overlapping metagenomic functions

## Graphical Abstract

**Figure:**
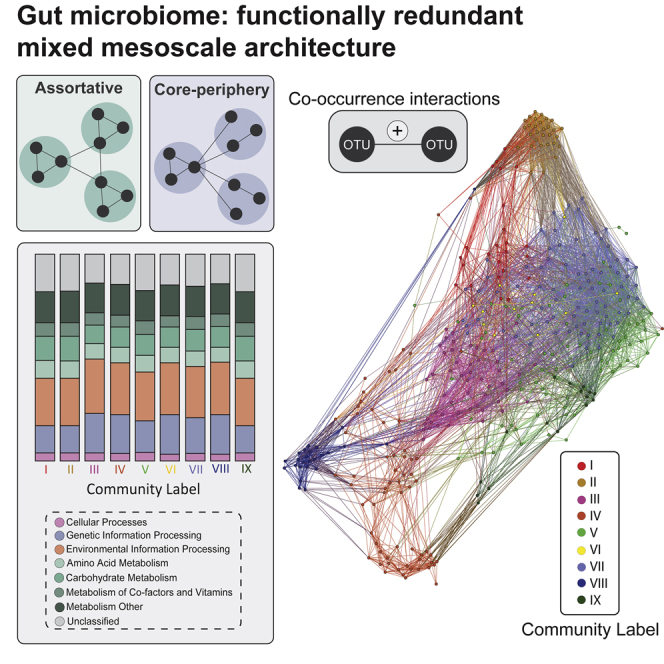

## Introduction

The human intestinal (gut) microbiome is a complex biological system, whose functions and metabolic processes are the product of multiple interactions between microbial operational taxonomic units (OTUs) (Zelezniak et al., 2015). These diverse interactions can arise from direct or passive mechanisms and may result in beneficial (commensal or mutualistic), neutral, or detrimental (competitive or parasitic) effects to all OTUs involved (Faust and Raes, 2012). Perturbations to microbial interactions may manifest as microbial dysbiosis and have been implicated in a number of pathologies including inflammatory bowel disease (Halfvarson et al., 2017), metabolic dysregulation (Sanz et al., 2014), and neuropsychiatric disorders (Jiang et al., 2015). Understanding the structure, function, and composition of the human microbiome has therefore become an active area of research (The Human Microbiome Project Consortium et al., 2012). Interest in the microbiome has also coincided with the adoption of network science in biology, offering an armory of conceptual and analytical tools to model microbial interactions.

**Figure 1:**
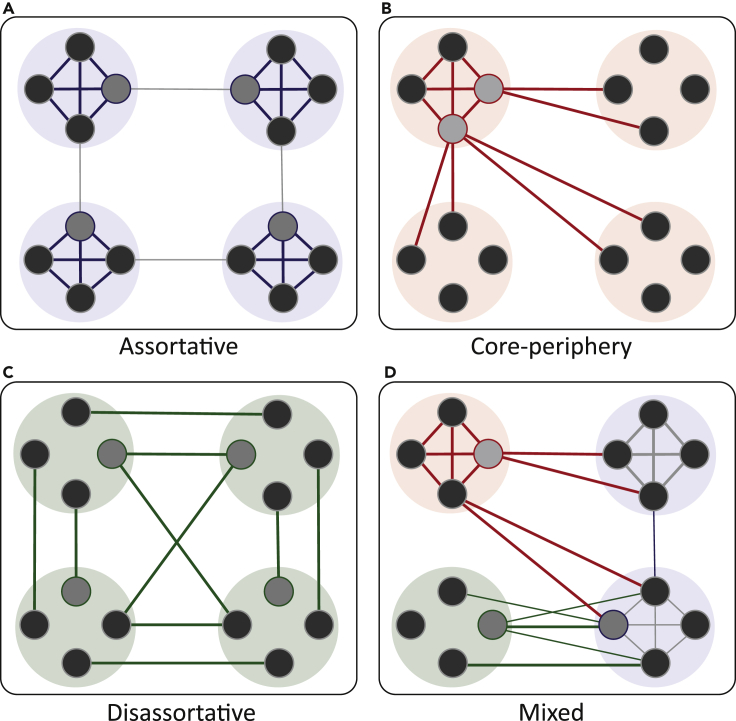
Representation of Possible Community Structures at the Mesoscale Level, Visualized as a Force-Directed Graph Layout (A–C) Mesoscale network structures can be (A) assortative, where the internal density of interactions (within community) exceeds the external density of interactions (between communities); (B) core-periphery, where there is a central core (connected to the rest of the network) and periphery (with minimal interactions); or (C) disassortative, where the external density of interactions (between communities) exceeds the internal density of interactions (within community). (D) Mesoscale structures can also occur simultaneously in the network, described as mixed or co-existing architectures (Betzel et al., 2018).

Using co-occurrence and co-exclusion relationships between individual OTUs, network-based approaches allow us to gain insight into the healthy and pathological properties of the microbiome, including organizational features that may contribute to system resilience or vulnerability (Baldassano and Bassett, 2016, Lozupone et al., 2012). Within the microbiome, OTUs are not expected to interact equally but to form smaller communities characterized by dense functional associations. An emerging approach to study the structure and function of the microbiome is therefore to define and characterize co-occurrence interactions at the mesoscale (community level). Mesoscale defines an intermediate level between that of individual OTUs and the whole microbiome. At the mesoscale, networks can exhibit different community structures, including assortative, disassortative, core-periphery, or mixed interactions (Betzel et al., 2018) (Figures 1A–1D). The dominant view emerging from existing work is that the human gut microbiome exhibits segregated and autonomous assortative communities, where OTUs sharing similar phylogenetic or functional properties have a tendency to preferentially cluster together. Evidence for a singular assortative structure in the microbiome has been observed in both empirical (Jackson et al., 2018) and computational modeling work.

Although previous work has provided important insights, the algorithms and heuristic approaches used to detect communities have certain design features such that they can detect only assortative communities. Modularity maximization, for example, is among the more popular community detection methods used in the field. However, this method is only capable of detecting groups of OTUs that are densely intra-connected and sparsely inter-connected: an assortative or modular community structure. Consequently, it is unclear whether the detected assortativity represents a methodological bias or is a reflection of the gut microbiome community structure. The current understanding of the microbiome architecture may therefore be too simplistic. Recent work on microbial interactions support the notion that the microbiome exhibits not only assortative, but also cores, peripheries, and disassortative communities. That is, the microbiome may exhibit densely connected “core” OTUs, whose metabolic and enzymatic processes exert a particularly beneficial role to the rest of the network, including the efficient transfer of nutrients, metabolites, or by-products (Ze et al., 2013). From an ecological perspective, a conserved core-periphery structure is consistent with the concept of a keystone guild. Keystone guilds—groupings of “core” or keystone OTUs—have been described as highly connected structures that exert a considerable influence on the structure and stability of the microbiome (Power et al., 1996, Banerjee et al., 2018). The ability to conform to multiple, co-existing network configurations may therefore reflect an ecological or evolutionary selective advantage to the microbiome. Specifically, this ability may be critical for the human microbiome, where environmental perturbations are frequently introduced, including dietary changes and/or antibiotic administration (Buffie et al., 2012).

Extending upon previous work, we studied the mesoscale architectures underpinning the human gut microbiome by applying the weighted stochastic block model (WSBM), a flexible generative algorithm for detecting community structure (Aicher et al., 2014). Unlike modularity maximization, where a network can only be partitioned into densely connected modular communities (Figure 1A), the WSBM also considers alternative patterns of connectivity that are less spatially compact (Figures 1B–1D). This provides the WSBM the flexibility to uncover multiple network structures beyond assortativity, including disassortative and core-periphery interactions between communities. The approach has recently been validated to partition and assign community network structures in models representing interactions between remote brain regions (Betzel et al., 2018, Faskowitz et al., 2018). Using the WSBM framework, we sought to (1) establish whether the human microbiome exhibits a unique or heterogeneous mesoscale network architecture; (2) understand the patterns of microbial co-occurrence relationships within and between communities; and (c) identify hub OTUs that may play a key role in supporting the mesoscale network structure or function. Based on emerging research, we hypothesized that the microbiome will exhibit both assortative and non-assortative structures, including core-periphery participation. From a taxonomic perspective, we expected closely related OTUs to form strong, assortative co-occurrence interactions. Non-assortative communities, however, are expected to exhibit greater taxonomic microbial diversity. Information on individual OTUs that exhibit specific network features, including high between- or within-community interactions, may provide additional insights into the key organizational principles supporting the microbiome.

## Results

### Applying the Weighted Stochastic Block Model to the Human Microbiome

**Figure 2:**
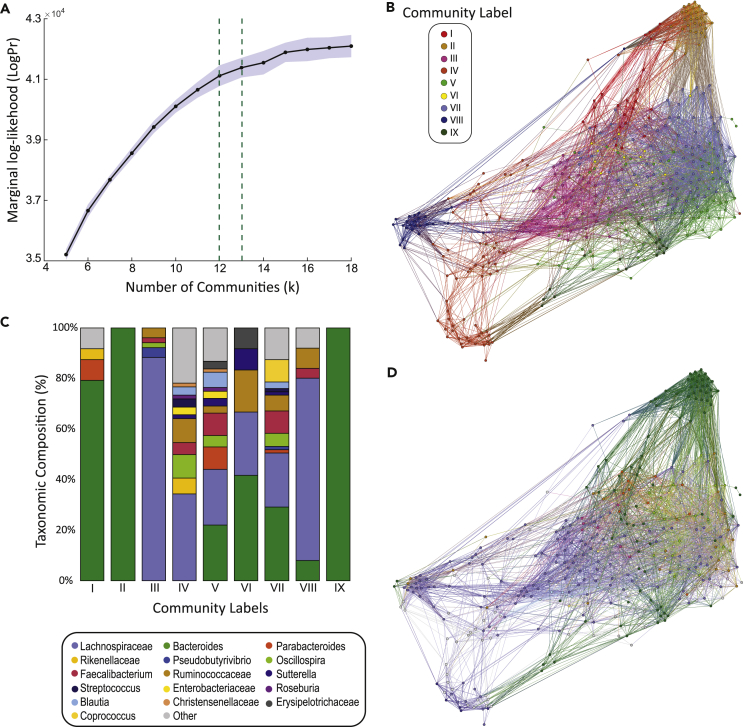
Bayesian Model Selection Using the Weighted Stochastic Block Model to Define the Microbial Interaction Network (A) We fit the WSBM algorithm across a number of models between *k* = 5 to *k* = 18, repeated 65 times each. The consensus partition, *k* = 12 (green, dashed), was achieved when mean marginal log likelihood, *LogPr* (blue, solid) plateaus. (B) Co-occurrence microbial interaction network, where each node represents an OTU and edges represent pairwise abundance relationships between them. The co-occurrence network has been reduced for visualization, representing 370 nodes and 4,854 edges at a Pearson correlation cutoff of r = 0.3. OTUs are colored according to community label. (C) Composition (%) of each community based on taxonomic assignment of the OTUs at the family/genus level. (D) The same co-occurrence network as seen in (B), but where OTUs have been colored according to taxonomic assignment at the family/genus level.

We created a microbial interaction network by fitting the WSBM to a dataset of 58 healthy human intestinal microbiomes, representing 370 nodes (OTUs). We then replicated the analyses in a large (n = 528), independent, and publicly available dataset. Using a Bayesian model optimization method (details in Transparent Methods), the microbiome network was partitioned into *k* = 12 communities, based on where the mean marginal log likelihood (a measure of model fitness) begins to plateau (Figure 2A; Figure S1 for replication). Note that we also performed a control analysis at *k* = 13, which yielded a similar consensus partition and mesoscale structure. At *k* = 12, we repeated the WSBM for 65 fits, each returning an independent partition assigning each OTU to one of 12 communities. The consistency of the community assignment was assessed using normalized mutual information (NMI) from the output of each WSBM partition. To test that the consistency of 65 detected community partitions were above chance level, the results were benchmarked against partitions that were achieved by fitting the WSBM to null networks. Microbiome community partitions were highly consistent with each other (NMI, 0.70 ± 0.02), whereas null networks had low consistency between each WSBM run (NMI, 0.38 ± 0.02). The NMI values obtained from comparing microbiome partitions were significantly higher than those obtained from the null (P < 0.001) (Figure S2). To estimate the impact of inter-subject variability in community detection, we performed a cross-validation leave-one-out (LOO) analysis (Figure S3). Results confirm that our group-level co-occurrence network was representative across 58 samples, with low inter-individual variability when compared with the original group-level matrix (as assessed by one minus Mantel's test statistic, μ = 0.02 ± 0.03) (Figure S3).

We subsequently reduced 65 repetitions from the microbiome dataset to a single consensus partition using a progressive median alignment method (Transparent Methods). The alignment algorithm detects consistent assignment to communities and reduces the complexity of the model if OTU assignment to any given community is low. Across 65 WSBM fits, three communities had low consistency in nodal assignment (i.e., OTUs assigned to these communities had equal preference for assignment with at least one other community). These communities were subsequently removed from the model, and OTUs were assigned to another community that they were more consistently aligned to. The final number of communities was therefore 9, ranging in size from 12 to 80 OTUs (Figure 2B). A complete list of the final consensus partition is presented in Table S1. We visualized the overlap between computational (community assignment) and taxonomic decompositions of the OTUs in Figures 2B–2D. As the WSBM learns from both the presence and weight of edges, thus far we have included both positive and negative interactions. Thus, the mesoscale analysis presents a coarse-graining of the network's overall architecture, accounting for the polarity of microbial interactions. However, given the specificity of our subsequent network-based statistics, we have opted to restrict our analyses to positive correlations only (thresholded at 0.4). This is a common approach in microbiome network studies to reduce statistical noise (Poudel et al., 2016, Jackson et al., 2018), as accurately interpreting the ecological significance of weak positive, and negative correlations is difficult in compositional data (i.e., relative abundance).

### Evidence for the Diversity of Mesoscale Architectures in the Microbiome

First, we sought to establish whether communities in the microbiome exhibit a single mesoscale structure or whether we find evidence of many different types. To answer this, we used community motif participation, an approach recently developed and validated to characterize mesoscale interactions between communities of brain regions (Betzel et al., 2018) (Transparent Methods). To estimate an OTU's motif participation, we first defined a community motif for every pair of communities as the average connection weight within and between those communities. Based on these average connection weights, the communities comprising each motif could be uniquely classified as assortative, disassortative, core, or periphery. Next, we mapped community-level classifications back onto the individual OTUs that comprise each community. Finally, for a given OTU, we calculated motif participation as the proportion of times its community participated in a given motif.

Results showed that the healthy human microbiome exhibited a preference for assortativity, with embedded core-periphery mesoscale architectures. No disassortative interactions were detected across all WSBM fits. We tested this preference for assortativity by calculating a maximum assortativity score, providing an indication of how often each community formed exclusive assortative motifs with all communities (i.e., 100% assortative interactions). Over 65 WSBM fits, maximum assortativity therefore represents the proportion of times the minimum within-community interactions exceeded the maximum between-community interactions for any pair of communities (Figure 1). On average, each OTU's community exhibited maximum assortativity 36.94 ± 8.45% of the time. We then calculated the proportion of times an OTU's community participated in any other type of structural motif beyond assortativity (e.g., a minimum of one pair of community interactions) and observed that as a whole network, core and peripheral interactions occurred 12.56 ± 16.60% and 13.25 ± 15.87% of the time, respectively. Importantly, comparative observations of assortative, core, and periphery motif interactions were also observed in our replication dataset (Figure S1). Replication of the above-mentioned findings in a distinct dataset (larger sample size, different age range, and broad ethnic diversity) suggests that the co-existence of complex architectures may be a ubiquitous property to the microbiome. The large variances observed between participation in core and periphery structures suggests each OTU's community may exhibit dissimilar patterns of mesoscale proportions, which we investigate below.

### Microbial Communities Exhibit Unique Mesoscale Signatures

The above findings corroborate previous analyses of microbiome mesoscale structure, identifying a mostly assortative organization (Baldassano and Bassett, 2016). Using the common community detection algorithm, modularity maximization, we further support the existence of a dominant assortative structure in this dataset (Figure S4). However, we also find evidence of non-assortative (primarily core-periphery) interactions. This suggests that a strictly assortative description may not fully characterize the diversity of mesoscale communities. We next sought to establish whether core-periphery interactions occurred uniformly between all nine communities (with each exhibiting a small degree of core-periphery interactions with at least one other community) or whether these observations are driven by a small subset of non-assortative interactions. We calculated the average number of times (over 65 WSBM fits) each community (Figure 2B) participated with other communities in a core or peripheral motif (Table 1). Results showed that most communities exhibited some degree of participation in non-assortative motifs (as shown by the participation in core and peripheral motifs) but were not uniformly distributed. To establish the significance of core and periphery mesoscale interactions, we used a permutation-based null model to create distributions representing the size and number of each community observed in the empirical dataset (Figure S5). When benchmarked against random community assignments, core motif participation was significantly higher in communities VII and VIII, whereas communities IV, V, and VI exhibited significantly higher participation in peripheral mesoscale motifs (Table 1, Figure 2B) (P < 0.05). These communities are characterized by a range of different OTUs classified at broader (phylum) and finer (family/genus) taxonomic levels, including four dominant bacterial phyla: *Firmicutes, Bacteroidetes, Actinobacteria, and Proteobacteria* (Figure 2C).

**Table 1:** Community-Level Network Statistics

Community Label	Number of Nodes	Core Participation (%)	Peripheral Participation (%)	Community Assortativity	Strength
I	24	0.04 ± 0.02	0.01 ± 0.02	0.08 ± 0.11	32.85 ± 5.30
II	30	0.11 ± 0.00	0.00 ± 0.00	0.56 ± 0.09^f^	43.99 ± 3.99
III	51	0.10 ± 0.04	0.01 ± 0.03	0.14 ± 0.07	37.10 ± 5.80
IV	64	0.02 ± 0.03	0.19 ± 0.16^c^	0.02 ± 0.10	17.50 ± 3.55
V	68	0.00 ± 0.01	0.27 ± 0.13^d^	−0.05 ± 0.05	23.86 ± 5.64
VI	12	0.02 ± 0.03	0.44 ± 0.10^e^	−0.01 ± 0.09	28.43 ± 4.54
VII	80	0.35 ± 0.17^a^	0.13 ± 0.14	0.03 ± 0.07	41.72 ± 7.15
VIII	25	0.31 ± 0.13^b^	0.08 ± 0.13	0.35 ± 0.17^g^	22.21 ± 4.32
IX	16	0.01 ± 0.01	0.03 ± 0.05	0.31 ± 0.10^h^	29.68 ± 3.44

Reported as mean ± SD.

a p Value = 2.68 × 10^−38^.

b p Value = 1.80 × 10^−08^.

c p Value = 8.68 × 10^−04^.

d p Value = 1.80 × 10^−16^.

e p Value = 5.66 × 10^−12^.

f p Value = 5.15 × 10^−40^.

g p Value = 8.92 × 10^−09^.

h p Value = 6.33 × 10^−05^.

To gain a more nuanced understanding of the mesoscale interactions between OTU communities, we additionally calculated nodal assortativity (normalized to the size of each community) (Faskowitz et al., 2018) and nodal strength (Rubinov and Sporns, 2010) (Transparent Methods) and mapped these onto community-level patterns (Table 1). In this context, positive assortativity coefficients (>0) should therefore be interpreted as exhibiting assortative interactions, whereas negative coefficients (<0) suggest this community may participate in disassortative interactions. Communities with assortativity coefficients of ∼0 do not exhibit strong assortative interactions as detected using the WSBM. As the WSBM algorithm can detect simultaneous mesoscale structures, it is important to note that a high assortativity coefficient does not preclude the existence of core or periphery participation (Table 1). Communities II, VIII, and IX exhibited the strongest preference for community assortativity, significantly higher than what would be expected based on the size of these communities (P < 0.05). These communities are characterized by robust co-occurrence interactions between taxonomically related OTUs: Community II and IX exclusively consisted of the OTUs related to the *Bacteroides* genus (Figure 2C). Although results have thus far suggested that assortative structures coincided with low microbial diversity, there are notable exceptions. Community VIII exhibited both assortative and core motifs and is composed of higher taxonomic microbial diversity at the genus level (Figure. 2C). The simultaneous existence of mesoscale architectures may suggest that assortativity is not a simple derivative of taxonomic relatedness. This hypothesis is further confirmed by an additional analysis assessing community assortativity as a product of taxonomic lineage. When OTUs were forced into communities based on their taxonomic classification at the phylum and family/genus levels (as per Figure 2D), we observed that mean nodal assortativity was lower compared with the WSBM-derived results.

### Determining the Functional Contribution of Communities

To gain insight into the functional contribution of each community, we used PICRUSt (Phylogenetic Investigation of Communities by Reconstruction of Unobserved States) Langille et al., 2013, a validated computational modeling approach that predicts a microbial community's metagenome from its 16S profile. An estimated functional contribution (i.e., to determine the degree to which OTUs contribute to specific metagenomic processes, %) was attributed at the OTU level. This information was used to estimate the functional contribution of each WSBM community. Results suggest that each community performs a broad repertoire of functional processes (Figure S6). Communities II and IX (composed exclusively of *Bacteroides*) exhibited the largest total functional contribution (%) and were particularly enriched for metabolic and genetic information processing. Results also highlight that some distinct functions were supported by specific communities. For example, processes facilitating cell motility (under *Cellular Processes*) are almost exclusively supported by community V (with a small contribution from community IV).

### Identification of Hubs

**Figure 3:**
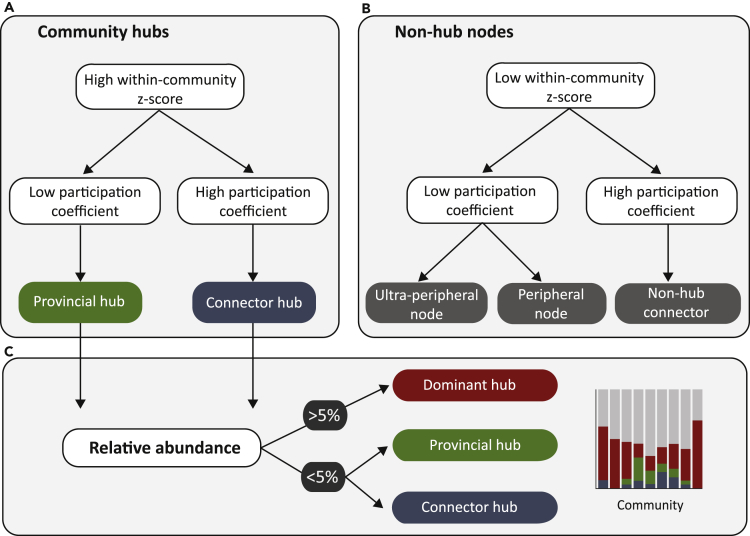
Classification of Individual OTUs Based on Within-and Between-Community Interactions and Relative Abundance (A) The classification of community hubs (provincial and connector) was based on high within-community *Z* score (>0). (B) The classification of non-module hubs (ultra-peripheral, peripheral, and non-hub connectors) was based on low within-community *Z* score (<0). (C) Provincial and connector hubs were further assessed in terms of their relative abundance (%) at the genus level. OTUs whose genus had >5% relative abundance in the microbiome were classified as “Dominant” hubs. The proportion of dominant, connector, and provincial hubs were finally visualized for each mesoscale community (Figure 4).

**Figure 4:**
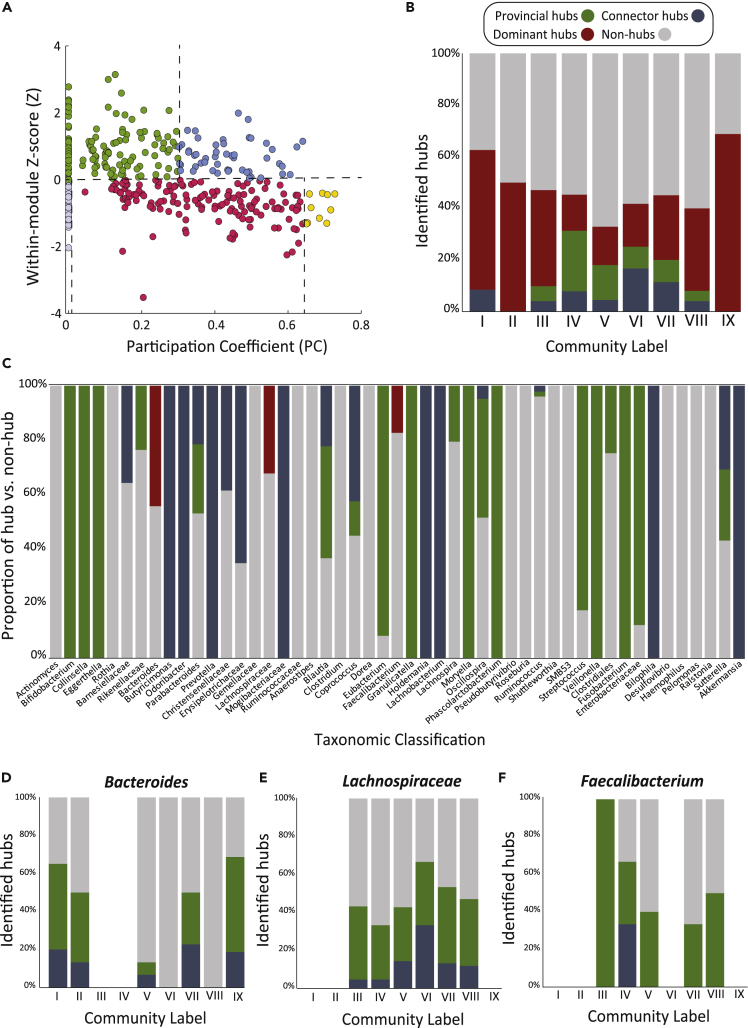
Role of Individual OTUs Based on Within- and Between-Community Co-occurrences (A) OTUs were partitioned into non-community hubs (*Z* < 0) or community hubs (*Z* > 0) based on their within-community *Z* score (Guimerà and Amaral, 2005). Non-community hubs were then grouped into ultra-peripheral (*PC* ≈ 0, mauve), peripheral (*PC* < 0.625, red), non-hub connectors (0.625 < *PC* ∠ 0.8, yellow), and non-hub kinless nodes (*PC* > 0.8, dark purple). Non-community hubs were then grouped into ultra-peripheral (PC ≈ 0, mauve), peripheral (PC < 0.625, red), non-hub connectors (0.625 < PC < 0.8, yellow) and non-hub kinless nodes (PC > 0.8, no detected OTUs). Community hubs were grouped into provincial hubs (0 < PC < 0.3, green), connector hubs (PC < 0.75, blue), or kinless hubs (PC > 0.75, no detected OTUs). (B–F) (B) Relative proportion (%) of provincial hubs (green), connector hubs (blue), dominant hubs (red), and non-hubs (gray) within each community and (C) when OTUs are assigned to their genus/family level, using the classification pipeline described in Figure 3. Relative proportion of provincial, connector, and non-hub nodes within each community for the dominant hubs (before reclassification), including all OTUs belonging to (D) *Bacteroides*, (E) *Lachnospiraceae,* and (F) *Faecalibacterium.*

Insights into the functional role of individual OTUs in the mesoscale network were then assessed by comparing interactions within (within-community *Z* score, *Z*_*i*_) and outside (participation coefficient, *PC*) WSBM-derived community labels (Figure 2B) (Guimerà and Amaral, 2005) (Transparent Methods). We used previously validated cutoffs as a broad guideline (Guimerà and Amaral, 2005) and subsequently assigned each OTU into one of the two broad classifications: non-hubs nodes and community hubs (Figures 3A, 3B, and 4A). At this stage, the classification of community hubs does not take into account the total abundance of the OTU within the microbiome. For example, a prevalent OTU might share many co-occurrences within the system exclusively by virtue of their sheer abundance (Banerjee et al., 2018). To make the distinction between dominant and true connector/provincial hubs, we assessed each candidate hub in terms of the relative abundance (%) of its corresponding genus (or family, where required). OTUs whose genus/family had a total relative abundance greater than 5% were re-classified as dominant hubs (Figure 3). Candidate OTUs with less than 5% total relative abundance in the microbiome were referred to as “true” provincial or connector hubs. We then visualized the proportion of “true” provincial and connector hubs, dominant hubs, and non-hub OTUs within each community (Figure 4B), as well as the proportion within each genus/family classification (Figure 4C). As the dichotomy between provincial and connector classifications within dominant hubs is informative, we also visualize the decomposition of provincial and connector hubs for the three dominant genera before they were re-classified as dominant hubs (Figures 4D–4F).

Dominant hubs including *Faecalibacterium*, *Bacteroides*, and *Lachnospiraceae* were ubiquitous to all communities (red in Figures 4B and 4C) but particularly enriched within communities I, II, and IX (54%, 50%, and 69%, respectively). Specifically, community IX had a high prevalence of dominant hubs that, before re-classification, exhibited characteristics of provincial hubs. True provincial hubs, characterized by high within- and low between-community interactions, were proportionately higher in communities IV and V (23% and 13%, respectively) (green in Figure 4A). For OTUs classified as connector hubs, a high proportion originated from core and peripheral communities VI and VII, exhibiting both strong within- and between-community interactions (Figure 4C) (Rubinov and Sporns, 2010). Community VIII exhibited both core and assortative mesoscale motifs but showed a low prevalence of provincial and connector hubs. This suggests that the ability of this community to participate in core-periphery structures may largely be driven by dominant hubs. Ultra-peripheral (mauve in Figure 4A) and peripheral (red in Figure 4A) OTUs were uniformly present across all communities.

## Discussion

This study highlights the co-existence of diverse mesoscale network architectures in the healthy human gut microbiome. Our results provided an alternative interpretation of the microbiome's network organization, in which it exhibited a principal mesoscale structure of assortativity, alongside the existence of clearly delineated cores and peripheries. Our innovative use of advanced network modeling methods provides significant advances to the understanding of the human microbiome, which, until now, has been conceptualized as a singular assortative community structure (Jackson et al., 2018).

The detection of both assortative and core-periphery architectures in the microbiome raises questions about the ecological and evolutionary selection processes that shaped this mesoscale formation. We first consider the notion that assortativity is an important feature for microbiome structure and function. Understanding the advantage of an assortative topology has long been a focus in the study of biological networks, including brain networks (Sporns and Betzel, 2016), protein interactions (Zhang et al., 2010), and complex systems in general (Noldus and Van Mieghem, 2015). Several studies support the notion that assortative communities emerge through environmental filtering (Levy and Borenstein, 2013), whereas others suggest that assortativity represents a selective advantage to increase network robustness and functional redundancy. However, the view that microbial communities exclusively function as independent modules is at odds with findings supporting the existence of integrative core-periphery assemblages in biological systems. This evidence supports the existence of “spatially distinct and highly connected” (core) structures as a means of facilitating efficient interactions between communities (Baldassano and Bassett, 2016, Banerjee et al., 2018, Mouquet et al., 2013). In the context of the human gut microbiome, these interactions may arise from complementary resource acquiring strategies, niche partitioning, or the transfer of resources, including metabolites. Groupings of highly connected hubs, analogous to a keystone guild, may therefore decrease the distance between disparate communities comprising the network and support greater microbial diversity and species survival (Sugihara and Ye, 2009). Although core-periphery structures promote interactions between spatially disparate communities, a network largely dependent on structures increases the risk of ecosystem collapse (Sugihara and Ye, 2009). For example, when a core community or hub is perturbed, significant downstream effects to the wider network may ensue, potentially resulting in microbial dysbiosis. The vulnerability of core-periphery structures is supported by computational work, demonstrating widespread perturbation of the healthy microbiome network following the simulated removal of few hub species (Baldassano and Bassett, 2016). That is, the co-existence of core-periphery and assortative mesoscale structures allow optimal interactions between OTUs while maintaining functional redundancy, respectively (Otokura et al., 2016).

The detection of diverse and co-existing network architectures is critical to understand the broad repertoire of biological phenomena underpinning OTU interactions. A prominent feature of the detected network architecture is the development of strong assortative co-occurrence interactions among closely related OTUs, a finding consistent with previous work (Chaffron et al., 2010, Jackson et al., 2018). This observation is in line with two converging hypotheses regarding the functional organization of the microbiome. The first hypothesis postulates that the assortative grouping of closely related OTUs may be a product of environmental filtering (Stuart, 2018), where challenges including nutrition availability and substrate conditions have favored specific microbial traits. These traits are likely shared among genetically related OTUs, explaining their co-occurrence in the network. It has also been suggested that closely related OTUs have overlapping functional roles, supporting a high degree of compensation or degeneracy (Fornito et al., 2015). However, individual OTUs will still need to maintain minimal niche differentiation, as competitive exclusion remains a dominant driver of co-occurrence patterns (Stuart, 2018). In our WSBM analysis, we observed two assortative communities (II and IX) characterized by strong within-community co-occurrences between OTUs of the abundant genus *Bacteroides.* Specifically, community IX was enriched with provincial hubs above what would be expected in a random null network, suggesting this community exhibits high local integration. *Bacteroides* are known to exhibit a high degree of functional flexibility in response to changing substrate conditions in the microbiome (Rios-Covian et al., 2017). This is supported in our predicted metagenomics assessment at both the OTU and community level, suggesting that *Bacteroides* indeed exhibits a diverse repertoire of functional capacity, alongside the largest overall contribution to microbiome processes (Figure S6).

The above-mentioned results are consistent with previous community detection methods, including those undertaken by modularity maximization algorithms (Jackson et al., 2018). However, our WSBM analyses have also detected coexisting mesoscale structures. Communities IV to VIII exhibited significant assortative, core, and/or periphery interactions and were further characterized by high taxonomic microbial diversity (Figures 2C and 2D). Evidence for “true” (those characterized by low relative abundance in the system) connector and provincial hubs suggest they have a central role in facilitating network integration (Fornito et al., 2015). This is in line with previous work (Ze et al., 2013) demonstrating that the growth and survival of many OTUs may rely on interactions with a few core OTUs. The existence of connector hubs in a core-periphery architecture also presents some risks, as perturbation to these nodes may result in whole-network destabilization.

Our mesoscale assessment thus far has distilled important organizational principles of the gut microbiome. Just as important, however, is to understand how network topological properties inform function. We performed a metagenomics assessment to discern generalizable functional patterns both within and between our WSBM-derived communities (Figure S6). Although we observed some evidence for a mono-functional system (i.e., distinct functions performed by single or few communities), the microbiome can be better described as a multi-functional system (Moya and Ferrer, 2016). That is, irrespective of taxonomic composition or diversity, each community contributes to a broad number of overlapping functions. Our findings are consistent with recent work (Heintz-Buschart and Wilmes, 2018, Zhu et al., 2015) and supports the gut microbiome's preference to maintain high functional redundancy and stability.

### Limitations of the Study

Community detection algorithms, like the WSBM, can offer powerful exploratory tools to elicit insights into the macroscopic (large-scale) and microscopic (e.g., microbe-microbe) patterns underpinning ecological networks. However, it is important to note that no algorithm can capture ground truth mesoscale architecture (Peel et al., 2017). Thus, the insights gained via our approach must be complemented with empirical data to allow a more direct assessment of the posited patterns of interactions and their functional roles. While the community partitions achieved by the WSBM draws parallels with known biological mechanisms, we have balanced the interpretation of the results to reflect these limitations. It is also important to consider the limitations associated with 16S rRNA datasets. Although OTUs were clustered based on the standard 97% similarity thresholds, compared with deeper sequencing techniques (i.e., shotgun metagenomics sequencing), our taxonomic classification lacks the sensitivity to detect all bacterial species. In addition, owing to the bias introduced by the PCR amplification step during the 16S rRNA gene procedure, not all microbes may be represented in the dataset. Therefore, potentially significant co-occurrence interactions between individual species or strains may not have been captured through 16S rRNA gene analysis. Future work adopting metagenomics shotgun sequencing (Almeida et al., 2019) is required to confirm our findings. Such analysis may also achieve a more nuanced level of specificity, as well as facilitate the analysis of directed networks. Directed networks may provide a more complete picture of biological interactions, including the directionality of trophic, metabolic, and signaling pathways of bacteria, fungi, and archaea.

In summary, our study highlights the importance of characterizing heterogeneous and co-existing mesoscale architectures to understand the ecology and functions of the human gut microbiome. We reported two independent microbial co-occurrence networks exhibiting core-periphery structures, supported by a backbone of strong assortative community interactions. These findings represent an advance over the current view of a modular and segregated microbial environment, presenting opportunities for research and clinical endeavors on the gut microbiome.

## Methods

All methods can be found in the accompanying Transparent Methods supplemental file.

## Data and Code Availability

Data and code related to this paper may be requested from the authors. Raw sequences are available online (https://www.ncbi.nlm.nih.gov/sra/PRJNA577760). The accession number for the dataset reported in this paper is NCBI: [PRJNA577760].

## Supplemental Information

Document S1. Transparent Methods and Figures S1–S6

Table S1. Consensus Assignment of OTUs to Community Label from the Original Microbiome Dataset, Related to Figure 2B–2D and Table 1
