# Supplementary material for: Co-existence of Network Architectures Supporting the Human Gut Microbiome
Source: iScience. 2019 Nov 21;22:380–91. doi: 10.1016/j.isci.2019.11.032 (PMC6911941; doi:10.1016/j.isci.2019.11.032)
Supplement: Document S1. Transparent Methods and Figures S1–S6 [file mmc1.pdf]

ISCI, Volume *22*

Supplemental Information

Co-existence of Network Architectures

Supporting the Human Gut Microbiome

Caitlin V. Hall, Anton Lord, Richard Betzel, Martha Zakrzewski, Lisa A. Simms, Andrew Zalesky, Graham Radford-Smith, and Luca Cocchi

Supplemental Material

Transparent Methods

Human microbiome datasets

The study was approved by the Human Research Ethics Committees of the Royal Brisbane and Women's Hospital (RBWH), Brisbane, Australia, and QIMR Berghofer Medical Research Institute, Brisbane, Australia. Written informed consent was obtained for all study participants in accordance with the Helsinki Declaration. The first dataset was generated from gut mucosal biopsies from 58 healthy Australian adults (53% female; mean age 52.3 ±12.5 years) who were attending the RBWH Department of Gastroenterology for a routine colonoscopy as part of a colorectal cancer family history screening (Zakrzewski et al., 2019). Gut mucosal biopsies were collected during the endoscope, transported on dry ice to the coordinating site, and stored at -80ºC until analyses. Exclusion criteria included previous diagnosis of a gastrointestinal disease or disorder, complex chronic illness, or pregnancy. In the event that a participant presented with abnormal colonoscopy or biopsy results, they were subsequently excluded. For the replication analysis, we accessed an independent and publicly available dataset, generated from 528 human fecal samples (Yatsunenko et al., 2012). The replication dataset included 326 individuals aged 0-17 years, and 202 adults aged 18-70 years, representing healthy Amerindians from the Amazonas of Venezuela, rural Malawians, and residents from USA metropolitan areas.

DNA extraction and 16S rRNA analysis

Following tissue homogenisation using tubes containing 1.4mm ceramic beads (Precellys Lysing Kit), DNA was extracted from samples using DNeasy Blood and Tissue Kit (QIAGEN). DNA was quantitated using Nanodrop 2000 (Thermo Scientific). PCR amplification was performed on the V3-V4 hypervariable region of the 16S rRNA gene, and sequenced on a MiSeq sequencer (Australian Genome Research Facility (AGRF), Brisbane, Australia). Sequence data were processed using Quantitative Insights Into Microbial Ecology (QIIME) software suite v1.9.1 using default settings. Low quality reads were filtered and removed, and remaining sequences were de-multiplexed using a custom script to reduce the possibility of a mismatch. Clustering occurred in a two-step process. Firstly, sequences were clustered into operational taxonomic units (OTUs) based on existing sequences in the Greengenes database v13.5 (97% identity threshold). Secondly, unclustered reads were clustered de-novo (97% identify threshold). The USEARCH package (UCLUST v8.0.1623) was used to assign the representative OTUs to taxonomic lineage, using the Greengenes database as a reference. Chimeric seed sequences as identified by UCHIME were removed, and singleton OTUs were discarded for all downstream analyses. Metagenomics functions were predicted using the PICRUSt algorithm (Langille et al., 2013), following the tutorial steps outlined elsewhere (http://picrust.github.io/picrust/). The functional profiles for each subject were deconvolved into OTU-specific functional profiles, which provides an estimation of how each OTU contributes to the sum of functional processes performed by a given community. The data analysis pipeline for the replication dataset, also using the Greengenes database for OTU clustering, has been described elsewhere [see Methods section of (Yatsunenko et al., 2012)].

Co-occurrence network construction

We prepared our microbial interaction network in accordance with Berry and Widder's (Berry and Widder, 2014) best practices for co-occurrence network construction. As OTU abundance tables are sparse (with values of 0 representing an absence of an OTU in a sample), we applied a threshold to filter out infrequent OTUs and included only those with the largest relative abundances (top 20%) across all 58 samples. For each pair of OTUs, we then computed the Pearson correlation coefficient across individuals to generate an undirected, weighted co-occurrence network between all pairs of OTUs. Accordingly, the matrix represented weighted pairwise interactions for 370 OTUs. The replication dataset (n = 528) was thresholded to include OTUs in the top 10% with the largest absolute abundances and subsequently represented weighted pairwise interactions for 1225 OTUs. The different threshold adopted for the replication dataset was selected to: (i) maintain a comparable number and microbial diversity as seen in the original dataset, and (ii) strike a balance between scientific rigor and computational limitations.

Applying the WSBM to the microbiome

The WSBM is a generative modelling approach used to detect and partition a network's nodes into a number of latent communities, *k*. The WSBM described here extends upon the classical stochastic block model by allowing the inclusion of weighted edges in sparse networks. This model eliminates the need to threshold a

matrix before input to the WSBM and thus, preserves information about the weights of co-occurrence and co-exclusion relationships. MATLAB (The Mathworks, USA) codes for the WSBM were sourced via http://tuvalu.santafe.edu/~aaronc/wsbm/. We applied the WSBM to create a weighted network of the human intestinal microbiome, using steps described in detail elsewhere (Aicher et al., 2014).

To infer community structure from the WSBM, the user is required to input *k,* a free parameter representing the total number of communities. The model selection technique we describe here optimizes *k* based on a value that maximizes the marginal log-likelihood and penalizes model complexity (Aicher et al., 2014). To achieve a representative partition from the WSBM, we varied the number of communities from $k = 5$ to $k = 18$ and repeated the algorithm 65 times. We inferred *k* by determining where the mean and maximum marginal log-likelihood plateaus. For values greater than $k = 12$, we observed a clear plateau, and selected the simplest model (**Figure. 2A**). Given the stochastic nature of the WSBM, we assessed the consistency of the resulting outputs using NMI (Cover, 2012). Specifically, we fit the WSBM to the same dataset for 65 additional independent repetitions at our selected model, $k = 12$, to create a new frequency prior. Our marginal log-likelihood values (50 internal trials over 65 fits) were highly consistent with the original fits, with an NMI of 0.99. These findings were replicated in our supplemental dataset, where we observed a plateau betwen $k = 12$ and $k = 13$ ($\boldsymbol{P} = 0.52$) (Supplemental Material, **Figure. S1**).

Consistency of community assignments

Each stochastic repetition of the WSBM returns a subdivision of non-overlapping groups of OTUs to one of 12 distinct communities. To assess if our detected partitions (n = 65) were significantly more consistent across trials to those obtained from a random network, we employed the *randmio_und* function from the BCT toolbox (Rubinov and Sporns, 2010), permuting the strength of connections while preserving the signed degree distribution. This resulted in 10 independent null networks. For each null, we then applied the WSBM for 65 fits, each with 5 internal trials (established as sufficient to ascertain consistent outputs for the null). The consistency between community assignments within each null was assessed using NMI. We then compared the consistency of the mean NMI scores (n = 650) with our mean MNI observed in our microbiome data.

Creating a consensus partition from WSBM fits

Due to the stochastic nature of the WSBM, the community assignment designated to each OTU may differ between runs. To ensure that each community was represented by a consistent identifier across all runs, we applied a progressive median alignment method using the software language R. This algorithm was developed in house and adapted from previous work (Lord et al., 2012). With our predefined *k*, the alignment script relabels communities to maximize consistency of community labels within each OTU across all runs. As such, the community structure within each subject is preserved, while consistency between labeling between runs is increased. After alignment, a single consensus partition that most accurately represents the 65 WSBM fits can be derived. We observed that, on average, OTUs at the individual level were assigned to the same cluster as the community consensus cluster 57% of the time (chance level 8.3%).

Visualization of the microbial network

The interactive platform Gephi (Version 0.9.2) was used to visualize the co-occurrence network, using the force atlas template (Bastian M., 2009). The network was reduced for visualization, using a cut-off of 0.3. This resulted in a network representing 370 nodes (OTUs) and 4854 edges (co-occurrence interactions). OTUs were colored according to community label, and taxonomic grouping at the family/genus level.

Community Motif Participation

We applied community morphospace analysis (Betzel et al., 2018) to classify community interactions into one of four motifs: assortative, disassortative, core, or periphery interactions. The community-level structures generated from 65 WSBM fits yielded a mesoscale description that mapped onto individual nodes (total n = 370, for the first dataset). Therefore, node *i*'s overall participation in each mesoscale structure was calculated as the number of times that an individual OTU's community interacted with other communities to form an interaction motif. Motif participation was averaged over 65 WSBM fits and expressed at the node (OTU), and community level. The significance of the detected mesoscale community structures was determined using permutation testing. For each community (n = 10), 650 null community partitions were created, comprising the same number and size of communities in the empirical dataset. A two-tailed test of the null hypothesis was performed with the resulting null distributions.

Nodal Assortativity

To determine nodal assortativity (Faskowitz et al., 2018), the weighted connectivity of node i (a_i) based on its assigned community (z) is compared to the maximum weighted connectivity to other communities. For each node, the weighted connection density to community m is defined as:

$$a_i(m) = \frac{1}{n_r} \sum_{j \in r} A_{ij} \,,$$

from which the nodal assortativity can be computed, as below:

$$a_i = a_i(z_i) - \max_{m \neq z_i} a_i(m).$$

Participation coefficient

The co-occurrence matrix was thresholded at 0.4, and network measures in the BCT toolbox (Rubinov and Sporns, 2010) were calculated to achieve insights at the global, mesoscale, and local scale. The participation coefficient (*PC*) measures the diversity of inter-community connections of node i, weighted by the importance of connections, and is defined as:

$$PC_i = 1 - \sum_{m \in M} \left(\frac{k_i(m)}{k_i} \right)^2,$$

where M is the number of communities, and $k_i\ (m)$ is the total connections between node *i* and all nodes in community *m*. A high value of *P* indicates that node i has strong connections with nodes outside its community, relative to connections within the community.

Within-community degree z-score

The within-community degree z-score, Z_i, measures the degree of intra-community connectivity of node i relative to other nodes in the community, and is defined as:

$$Z_i = \frac{k_i(m_i) - \bar{k}\ (m_i)}{\sigma^{k(m_i)}},$$

where m_i is the community containing node i, $k_i(m_i)$ is the within-community degrees between node i and all other nodes, $\bar{k}(m_i)$ and $\sigma^{k(m_i)}$ are the mean and standard deviation of the within-community degrees distribution. Higher values of Z_i (> 0) indicates node i has strong connectivity within its own community.

Nodal Strength

Nodal strength is defined as the sum of weighted edges connected to that node:

$$s_i = \sum_j w_{ij} \,,$$

where w_{ij} is the weight of edges between *i* and *j*.

Functional cartography measures

We broadly classified each node (OTU) into one of seven possible roles according to their participation coefficient and within-community Z scores, using previously validated cut-offs as a guideline (Guimerà and Amaral, 2005). This was achieved in a two-step process, involving:

1. *Classification of non-hub nodes (Z_i<0):*
   a) Ultra-peripheral nodes, characterized by exclusive intra-community connections (*P* ≈ 0);
   b) Peripheral nodes, characterized by moderately high intra- and low inter-community connections (*PC* <0.625);
   c) Non-hub connectors, where 62.5 – 80% of connections are within the community (0.625 < *PC* < 0.8);
   d) Non-hub kinless nodes, nodes that cannot be associated with any single community (*PC* >0.8).
2. *Classification of community hubs (Z_i>0)*
   e) Provincial hubs, characterized by high intra- and low inter-community connections (0 < *PC* < 0.30)
   f) Connector hubs, characterized by moderately high inter- and high intra-community connections (*PC* < 0.75)
   g) Kinless hubs, similar to non-hub kinless nodes, are not clearly associated with any single community (*PC* > 0.75).

Supplemental Figure Legends

Figure S1. Bayesian model selection using the WSBM on a replication dataset, Related to Figure 2A. We repeated the WSBM algorithm for our replication dataset (n = 528), across a number of models between *k* = 10 to *k* = 16 (repeated 10 times each). Using the Bayesian model optimization method (details in Transparent Methods), the replication dataset was partitioned into *k* = 12 communities (green, dashed), given that the mean marginal log-likelihood first plateaued at this point (**Figure. S1.**). As observed in the main text, assortativity was the dominant mesoscale community structure detected, alongside observations of core and peripheries. Again, no disassortative mesoscale structures were detected in the replication dataset. In the main text, we demonstrated maximum assortativity was evident 37% of the time across 370 OTUs. We reproduced this analyses and found that the results corroborate those of the main text. We observed maximum assortativity 27% of the time across 1225 OTUs. When calculating the proportion of times a node participated in non-assortative motifs, we observed core and peripheral interactions 3.84 ± 4.0, and 2.00 ± 3.40 percent of the time, respectively. These supplementary findings bolster the findings from the main text, and suggest that our results can be observed in two, independent microbial co-occurrence datasets. The replication dataset demonstrated that the human microbiome can be described as an assortative network with co-existing and nested non-assortative mesoscale architectures, as detected by the WSBM.

Figure S2. Consistency of detected communities as benchmarked against null models, Related to Table 1. The consistency of each community detection partition was assessed by calculating the NMI between each possible combination of partitions (total = 65), and benchmarked against 10 null models (each with 65 null partitions) generated from randomized networks. The community partitions observed in the microbiome were highly consistent (0.70 ± 0.02, red dashed line), and significantly different when compared to 650 random partitions generated from rewired null networks (NMI, 0.38 ± 0.02, blue).

Figure S3. Individual contribution to the group-level co-occurrence network, Related to Figure 2B&D. To derive information about single-subject community variability and confirm that our WSBM analyses is representative of the group, we performed a cross-validation analysis based on the leave-one-out (LOO) approach. In the LOO strategy, each subject's contribution to the resultant network structure is estimated by leaving that subject out and re-estimating group-level co-occurrence coefficients. To assess the similarity between the original and the n-1 networks, we used Mantel's test statistic, where a value of 1 indicates high similarity between networks (Mantel, 1967). We then assessed the average variability of each subject's contribution (μ) for each pairwise OTU interaction by subtracting the subject with the group-level correlation matrix. To test that our LOO strategy did not result in changes to WSBM-derived mesoscale architectures, we selected subjects with the greatest variability (>1 STD from the mean, subjects 13, 22, 33, 40, and 45) and re-fit these matrices to the WSBM 65 times at k = 12. The resultant consensus partition obtained from each of the networks (mean NMI = 0.90) were highly consistent with the original group-level analyses.

Figure S4. Comparing WSBM with an alternative community detection approach, Related to Figure 2B&D. To assess the validity of our findings, we assessed the putative modular structure using an alternative algorithm: The deterministic spectral modularity maximization algorithm (sites.google.com/site/bctnet/; function modularity_und). Modularity maximization detected five assortative communities that overlapped with the nine detected via the WSBM. Moreover, the mean nodal assortativity was highly consistent between algorithms (**R** = 0.83, **P** <0.001). These results are consistent with previous work comparing the WSBM to modularity maximization (Faskowitz et al., 2018). We here iterate that, contrary to the WSBM, modularity maximization can only detect assortative structures. Our comparative results suggest that the WSBM provides greater sensitivity to the detection of structures that do not conform to the strict assortative description required by other methods.

Figure S5. Proportions of mesoscale motif participation (%) under a permutation-based null model, Related to Table 1. To test that our detected assortative, core, and peripheral mesoscale motif interactions were greater than chance, we created 650 random partitions of community assignment (blue) and re-calculated the percentage of motif participation for a community of the same size and number. We benchmarked our observed community motif interactions (red, dashed) against the null community partitions (blue) and determined significance at ***P*** < 0.05. (A) Significant core motif interactions were observed within communities VII, and VIII. (B) Significant periphery motif interactions were observed within communities IV, V, and VI. (C) Significant assortative motif interactions were observed within communities II, VIII, and IX.

Figure S6. Predicted functional contributions for each community based on PICRUSt (Phylogenetic Investigation of Communities by Reconstruction of Unobserved States) (Langille et al., 2013), Related to Figure 2. Results showed that each community has the ability to perform a broad repertoire of functions.

Supplemental References

Aicher, C., Jacobs, A. Z. & Clauset, A. (2014). Learning latent block structure in weighted networks. *Journal of Complex Networks,* 3**,** 221-248.

Bastian M., H. S., Jacomy M. (2009). Gephi: an open source software for exploring and manipulating networks. *International AAAI Conference on Weblogs and Social Media*.

Berry, D. & Widder, S. (2014). Deciphering microbial interactions and detecting keystone species with co-occurrence networks. *Frontiers in microbiology,* 5**,** 219-219.

Betzel, R. F., Medaglia, J. D. & Bassett, D. S. (2018). Diversity of meso-scale architecture in human and non-human connectomes. *Nature Communications,* 9**,** 346.

Cover, T. M., Thomas, J.A. (2012). *Elements of information theory.*, John Wiley & Sons.

Faskowitz, J., Yan, X., Zuo, X.-N. & Sporns, O. (2018). Weighted Stochastic Block Models of the Human Connectome across the Life Span. *Scientific Reports,* 8**,** 12997.

Guimerà, R. & Amaral, L. a. N. (2005). Cartography of complex networks: modules and universal roles. *Journal of statistical mechanics (Online),* 2005**,** nihpa35573-nihpa35573.

Langille, M. G. I., Zaneveld, J., Caporaso, J. G., Mcdonald, D., Knights, D., Reyes, J. A., Clemente, J. C., Burkepile, D. E., Vega Thurber, R. L., Knight, R., Beiko, R. G. & Huttenhower, C. (2013). Predictive functional profiling of microbial communities using 16S rRNA marker gene sequences. *Nature Biotechnology,* 31**,** 814.

Lord, A., Horn, D., Breakspear, M. & Walter, M. (2012). Changes in Community Structure of Resting State Functional Connectivity in Unipolar Depression. *PLOS ONE,* 7**,** e41282.

Mantel, N. (1967). The Detection of Disease Clustering and a Generalized Regression Approach. *Cancer Research,* 27**,** 209.

Rubinov, M. & Sporns, O. (2010). Complex network measures of brain connectivity: uses and interpretations. *Neuroimage,* 52**,** 1059-69.

Yatsunenko, T., Rey, F. E., Manary, M. J., Trehan, I., Dominguez-Bello, M. G., Contreras, M., Magris, M., Hidalgo, G., Baldassano, R. N., Anokhin, A. P., Heath, A. C., Warner, B., Reeder, J., Kuczynski, J., Caporaso, J. G., Lozupone, C. A., Lauber, C., Clemente, J. C., Knights, D., Knight, R. & Gordon, J. I. (2012). Human gut microbiome viewed across age and geography. *Nature,* 486**,** 222.

Zakrzewski, M., Simms, L. A., Brown, A., Appleyard, M., Irwin, J., Waddell, N. & Radford-Smith, G. L. (2019). IL23R-Protective Coding Variant Promotes Beneficial Bacteria and Diversity in the Ileal Microbiome in Healthy Individuals Without Inflammatory Bowel Disease. *J Crohns Colitis,* 13**,** 451-461.

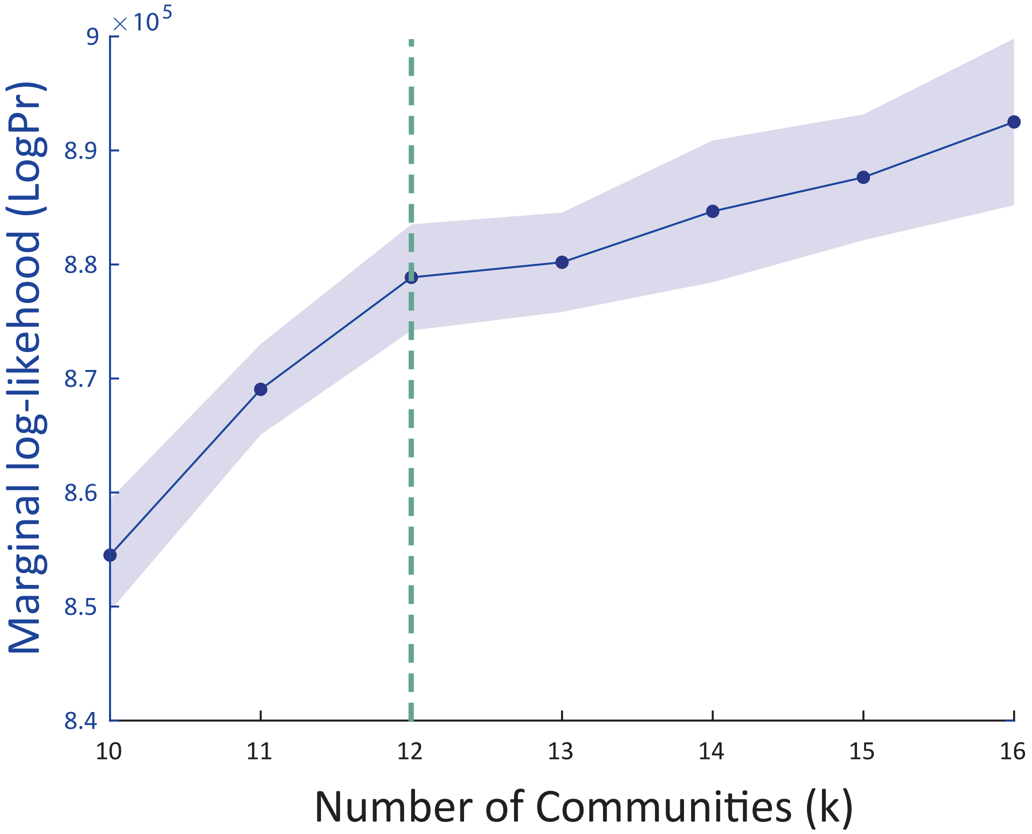

Supplemental Figure 1

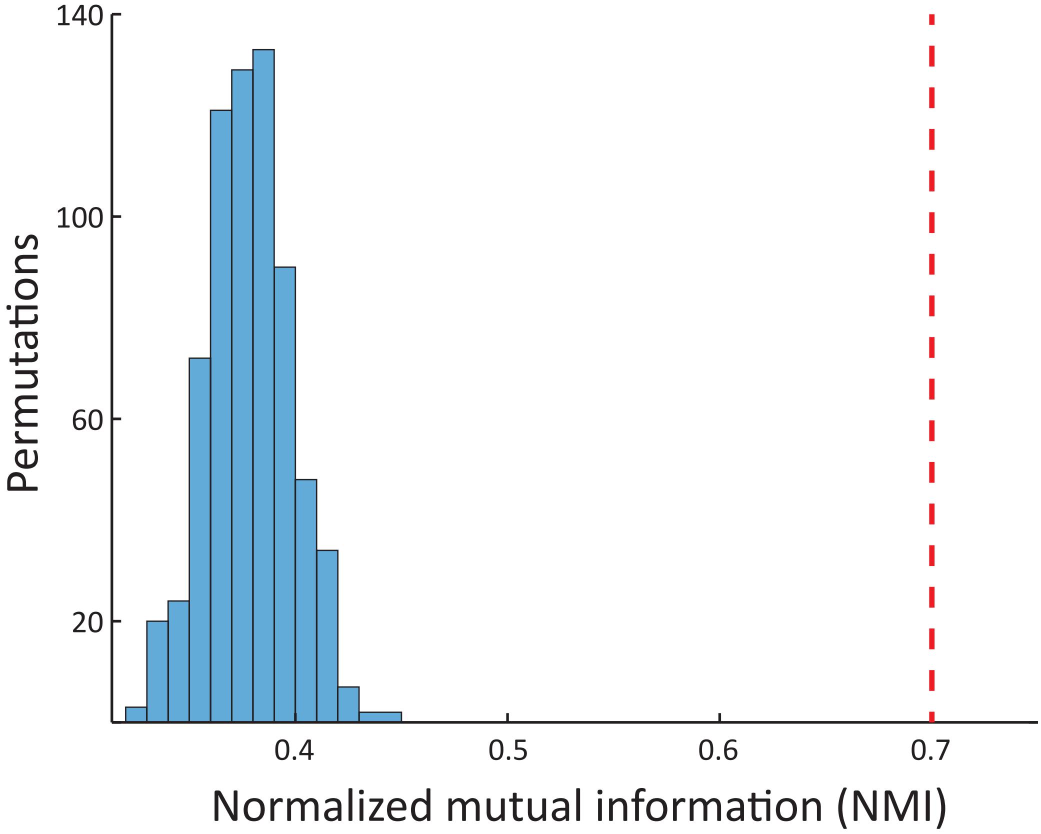

Supplemental Figure 2

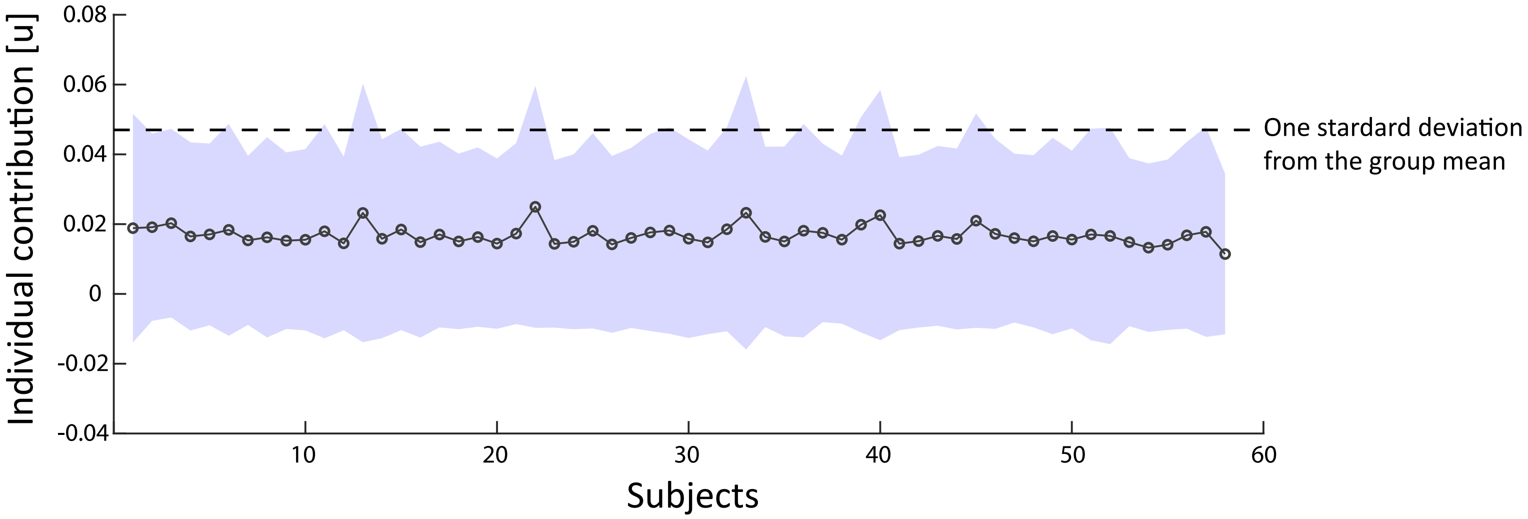

Supplemental Figure 3

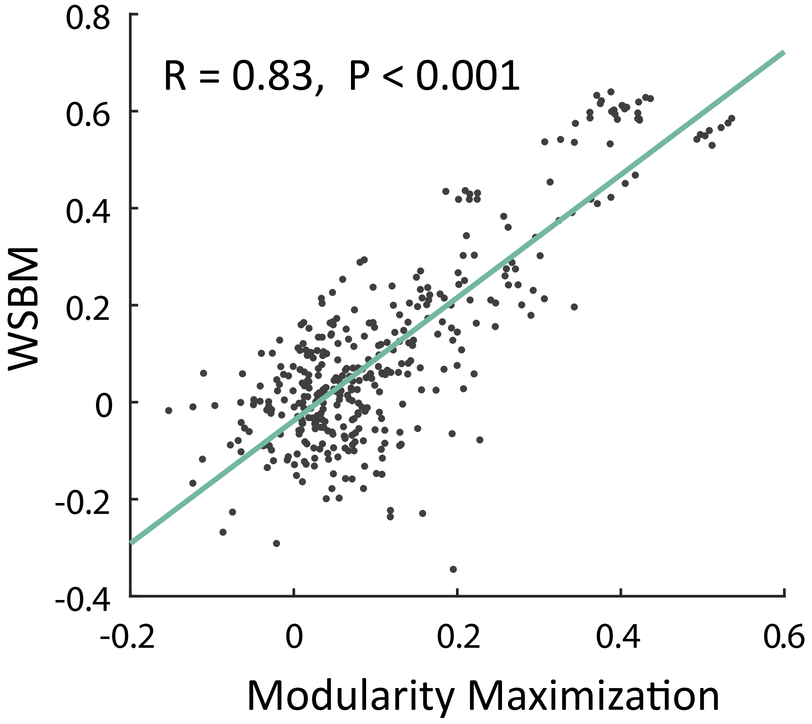

Supplemental Figure 4

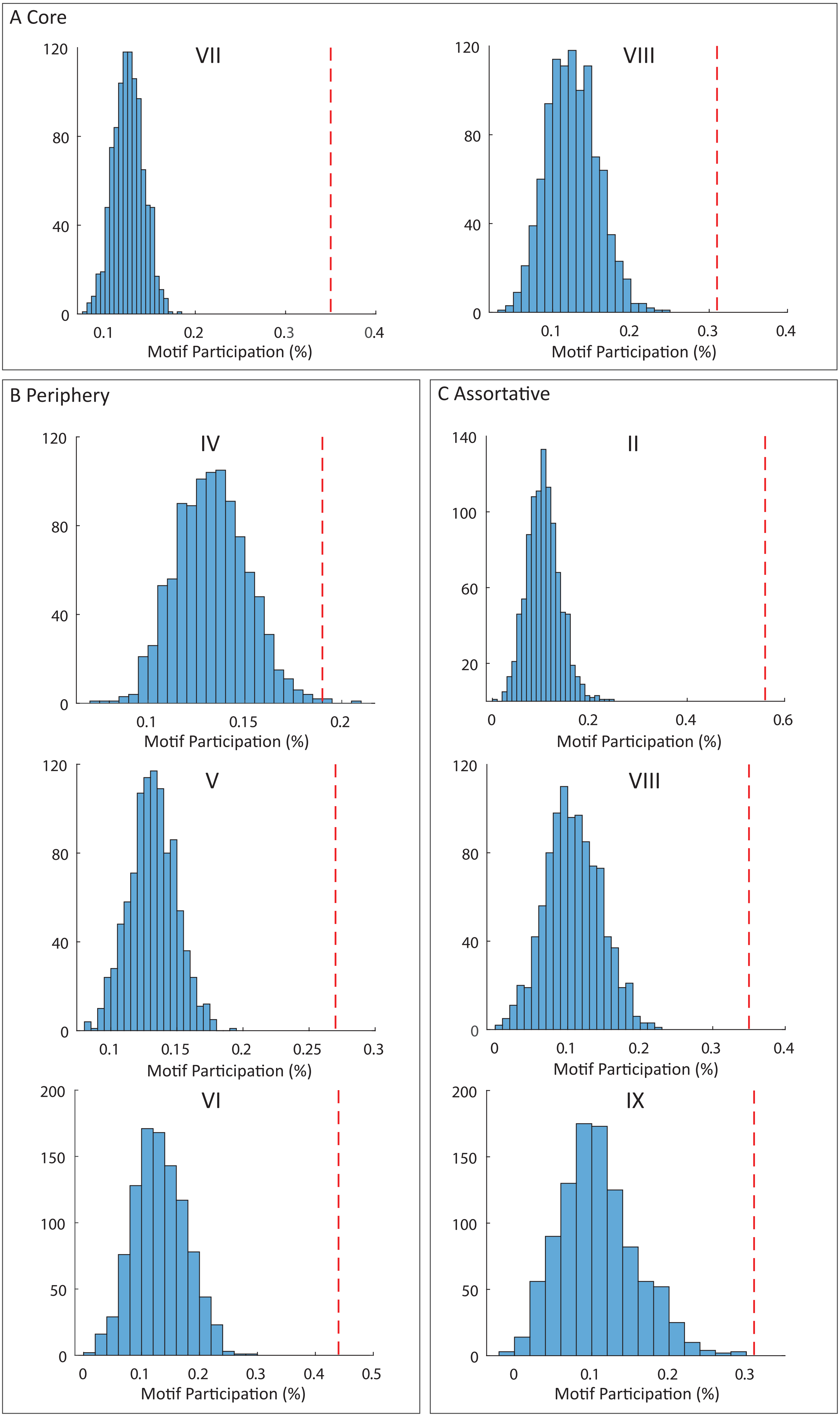

Supplemental Figure 5

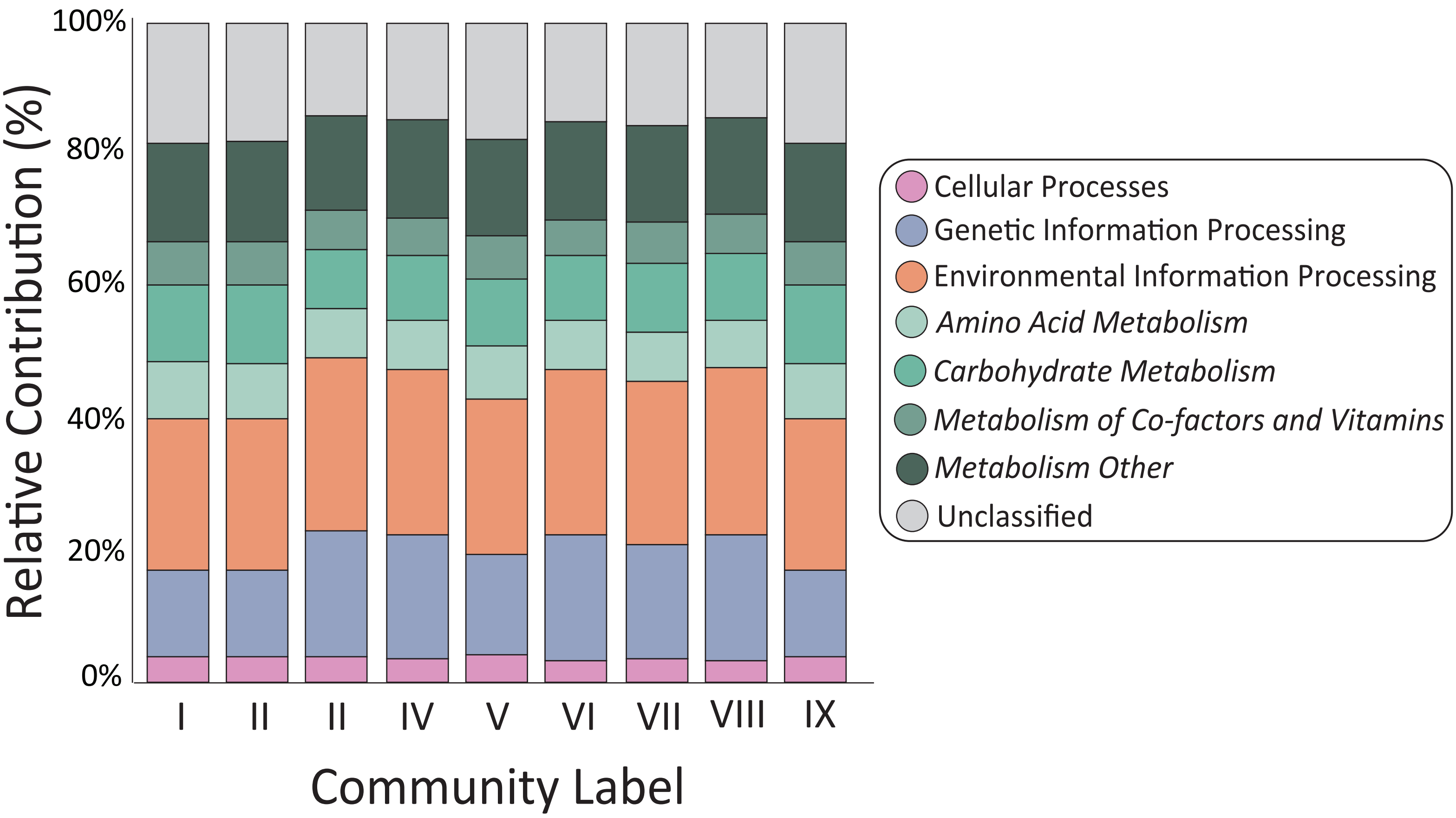

Supplemental Figure 6
